# A therapeutic antibody targeting annexin-A1 inhibits cancer cell growth in vitro and in vivo

**DOI:** 10.1038/s41388-023-02919-9

**Published:** 2024-01-10

**Authors:** Hussein N. Al-Ali, Scott J. Crichton, Charlene Fabian, Chris Pepper, David R. Butcher, Fiona C. Dempsey, Christopher N. Parris

**Affiliations:** 1 Anglia Ruskin University, School of Life Science, Faculty of Science and Engineering, East Road, Cambridge, CB1 1PT UK; 2 Medannex Ltd, 1 Lochrin Square, 92-98 Fountainbridge, Edinburgh, Scotland EH3 9QA UK; 3 Brighton and Sussex Medical School, Medical Research Building, Falmer, Brighton, BN1 9PX UK

**Keywords:** Cancer, Drug development

## Abstract

In this study we conducted the first investigation to assess the efficacy of a novel therapeutic antibody developed to target annexin-A1 (ANXA1). ANXA1 is an immunomodulatory protein which has been shown to be overexpressed in, and promote the development and progression of, several cancer types. In particular, high ANXA1 expression levels correlate with poorer overall survival in pancreatic and triple-negative breast cancers, two cancers with considerable unmet clinical need. MDX-124 is a humanised IgG1 monoclonal antibody which specifically binds to ANXA1 disrupting its interaction with formyl peptide receptors 1 and 2 (FPR1/2). Here we show that MDX-124 significantly reduced proliferation (*p* < 0.013) in a dose-dependent manner across a panel of human cancer cell lines expressing ANXA1. The anti-proliferative effect of MDX-124 is instigated by arresting cell cycle progression with cancer cells accumulating in the G_1_ phase of the cell cycle. Furthermore, MDX-124 significantly inhibited tumour growth in both the 4T1-luc triple-negative breast and Pan02 pancreatic cancer syngeneic mouse models (*p* < 0.0001). These findings suggest ANXA1-targeted therapy is a viable and innovative approach to treat tumours which overexpress ANXA1.

## Introduction

Annexin-A1 (ANXA1) is a member of the annexin protein superfamily that bind to acidic phospholipids in a calcium-dependent manner. ANXA1 is composed of a ‘core’ domain containing several repeating motifs and a unique ‘N-terminal’ domain of approximately 43 residues in length. The core domain has a high degree of homology with other annexin family members and facilitates calcium-mediated binding to cell membranes, whilst the N-terminal domain confers many of the functional properties of ANXA1 [1]. ANXA1 is normally localised in the cytoplasm, however it can be secreted into the extracellular environment where it can modulate cell behaviour in an autocrine, paracrine or juxtacrine manner through the activation of formyl peptide receptors 1 and 2 (FPR1/2) [2]. Once secreted, ANXA1 is also susceptible to proteolytic cleavage generating fragments of the protein with differing biological activity [3, 4].

Much of the early literature describes ANXA1 as a pro-resolving mediator of inflammation [5]. However, more recently, a growing number of studies have suggested that ANXA1 can promote tumour development and progression [6, 7]. ANXA1 expression has been found to be increased in cancerous tissue versus adjacent or matched non-cancerous tissue in several tumour types including pancreatic [8], colorectal [9], lung [10] and gastric cancer [11], with high levels of ANXA1 expression often being associated with poor patient prognosis and lower overall survival [8, 11, 12]. In addition, high levels of ANXA1 expression are consistently observed in triple-negative breast cancer (TNBC), compared with other breast cancer subtypes [13–15], suggesting that it might be a promising therapeutic target in these tumours.

The overexpression of ANXA1 by cancer cells has been demonstrated to increase cell proliferation [16], angiogenesis [17], migration/ invasion [18] and drug resistance [19]. ANXA1 has also been observed to influence immune cells and the tumour microenvironment by enhancing regulatory T-cell function [20], enhancing the polarisation and activation of M2 tumour-associated macrophages [21], suppressing dendritic cell activation and impairing CD8^+^ T-cell anti-tumour immunity [22]. Furthermore, ANXA1 is a key component of tumour-derived extracellular vesicles promoting migration, invasion and angiogenesis [23] and is secreted by cancer associated fibroblasts increasing cancer stem cell generation [24].

In contrast, some studies have found downregulation of ANXA1 expression in cancers such as cervical and thyroid [25, 26]. Moreover, there are some subtypes of breast cancer where ANXA1 expression is reduced, with several studies reporting decreased ANXA1 expression in ductal carcinoma in situ and invasive ductal carcinoma compared to normal and benign tissues [13, 27].

These conflicting data suggest that the expression and function of ANXA1 as a tumour suppressor or a tumour promoter is regulated in a tissue and tumour-specific manner. There have been suggestions that the role of ANXA1 may be specific to each tumour type due to post-translational modifications of the protein impacting expression across a range of cell types or cancer indications [28]. As a result, further studies are needed to investigate whether the inhibition of ANXA1 could provide a new therapeutic approach in certain major tumour types.

The purpose of this study was to determine whether MDX-124, a humanised monoclonal antibody targeting ANXA1 [29], could impact cancer cell growth using in vitro and in vivo models.

## Results

A panel of human cancer cell lines representing several major tumour types including breast, pancreatic, ovarian, colorectal and lung was chosen and used throughout this study (Supplementary Table 1).

### Expression and cellular localisation of ANXA1 in cancer cell lines

**Fig. 1:**
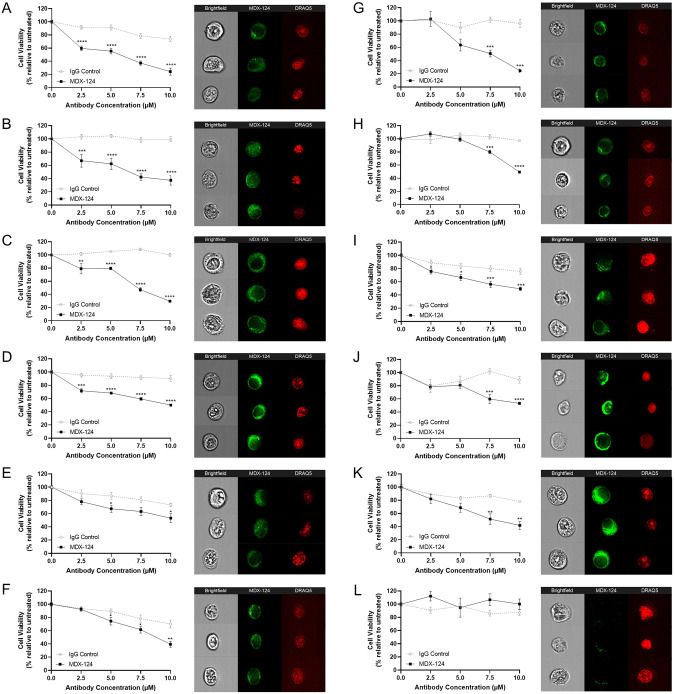
MDX-124 significantly reduces cancer cell proliferation which is associated with ANXA1 expression. **A** MCF-7, **B** HCC1806, **C** MDA-MB-231, **D** BxPC-3, **E** MIA PaCa-2, **F** A2780, **G** A2780ADR, **H** A2780cis, **I** Caco-2, **J** NCI-H69/CPR, **K** A549 and **L** COR-L23 cancer cell lines were treated for 72 h with either MDX-124 or IgG1 isotype control (2.5, 5, 7.5 or 10 µM). Loss of cell viability indicated by reduced metabolic activity was measured by MTT assay. Data are presented as the mean ± SEM of at least 3 independent experiments. Statistical significance calculated via Mann–Whitney *U*-test and indicated by ****p < 0.0001, ***p < 0.001, **p < 0.01 and *p < 0.05. ANXA1 expression and cellular localisation for each cell line measured by IFC. Left-hand panel shows a brightfield image. Central panel shows ANXA1 expression and cellular localisation as measured by fluorescently tagged MDX-124. Right-hand panel shows nuclear staining using Draq5.

Imaging flow cytometry was used to determine the level of expression and cellular localisation of ANXA1 in representative cancer cell lines. ANXA1 expression was observed in all cancer cell lines assessed except for COR-L23 lung cancer cells (Fig. 1). Furthermore, this was also shown to be localised in the cytoplasmic compartment and plasma membrane with little to no observable nuclear expression present (Fig. 1). Cell images are presented in multispectral form with the first column representing a brightfield image, the middle column showing staining of ANXA1, and the final column showing nuclear staining.

### MDX-124 significantly reduces in vitro cancer cell proliferation

MTT assays were used to determine whether MDX-124 could induce a functional anti-proliferative response across the panel of cancer cells by targeting ANXA1. MDX-124 caused a significant reduction in cellular proliferation in all breast, pancreatic, ovarian and colorectal cancer cell lines expressing ANXA1 when compared to an IgG1 isotype control antibody (Fig. 1, *p* < 0.05). This anti-proliferative activity occurred in a dose-dependent manner across the concentration range evaluated (0–10 µM).

Of the breast cancer cell lines assessed, inhibition of cell proliferation was most pronounced in the MCF-7, HCC1806 and MDA-MB-231 cell lines with 10 µM MDX-124 reducing viability by more than 50% versus IgG1 control-treated cells after 72 h (Fig. 1A–C). In the tamoxifen-resistant MCF-7/TAMR7 cell line, inhibition of cell viability, while significantly different from the IgG1 isotype control, was less pronounced at 21% compared to IgG1 control-treated cells (Supplementary Fig. 1A). Similarly, MDX-124 significantly inhibited proliferation of BxPC-3 (Fig. 1D), MIA PaCa-2 (Fig. 1E) and PANC-1 (Supplementary Fig. 1B) pancreatic cancer cell lines by 40%, 20% and 34% respectively versus IgG1 control-treated cells.

Ovarian cancer cell lines were also significantly impacted by MDX-124 with cell growth reduced by 31% (A2780), 48% (A2780cis) and 72% (A2780ADR) respectively versus IgG1 control (Fig. 1F–H). Across the colorectal cancer cell lines, MDX-124 (10 µM) caused a significant reduction in cell proliferation in Caco-2 cells (Fig. 1I), as well as HCT116 and SW480 cells (Supplementary Fig. 2B, C) versus IgG1 control-treated cells. Both NCI-H69/CPR and A549 lung cancer cells were found to be responsive to MDX-124 treatment, reducing cell proliferation by 36% and 37% respectively when compared to IgG1 control-treated cells (Fig. 1J, K). Conversely, incubation with either MDX-124 or the IgG1 control had no discernible anti-proliferative activity in COR-L23 (Fig. 1L) and COR-L23.5010 cisplatin-resistant (Supplementary Fig. 1E) lung cancer cell lines.

Overall, these data indicate that the anti-proliferative effect of MDX-124 is associated with ANXA1 expression, as cell lines responsive to MDX-124 treatment all expressed ANXA1, whereas a non-expressing lung cancer cell line (COR-L23) exhibited no response to MDX-124.

### Increased ANXA1 expression in tumours correlates with poorer survival in pancreatic cancer

Kaplan–Meier plots were generated using ANXA1 RNA-seq gene expression and probability of survival data from The Cancer Genome Atlas (TCGA) to evaluate any link between ANXA1 RNA expression and patient survival probability in breast, pancreatic, ovarian, colorectal and lung cancer. The results shown here are in whole or part based upon data generated by the TCGA Research Network: https://www.cancer.gov/tcga.

Within the pancreatic patient cohort (*n* = 91) those in the upper quartile of ANXA1 expression had a significantly poorer survival probability than those in the lower quartile (Supplementary Fig. 2A). Although patients in the breast (*n* = 1194), ovarian (*n* = 153) and lung (*n* = 538) cancer cohorts all trended in a similar manner to those in the pancreatic cohort, none of these reached statistical significance (Supplementary Fig. 2B–D). Interestingly, in the colorectal cancer cohort, the probability of survival trended towards higher ANXA1 expression being associated with a survival advantage versus those in the lower quartile of expression, although this was not statistically significant (Supplementary Fig. 2E).

### MDX-124 alters cell cycle distribution but does not induce apoptosis

To identify the mechanism by which MDX-124 inhibits cancer cell proliferation, both an annexin V apoptosis assay and cell cycle analyses were performed. Following 72 h exposure to MDX-124 (5 µM) there was no significant difference in the percentage of MCF-7 or Caco-2 cancer cells in early or late-stage apoptosis versus untreated control cells (Supplementary Fig. 3). This indicates that the anti-proliferative activity of MDX-124 is not mediated via an induction of apoptosis.

**Fig. 2:**
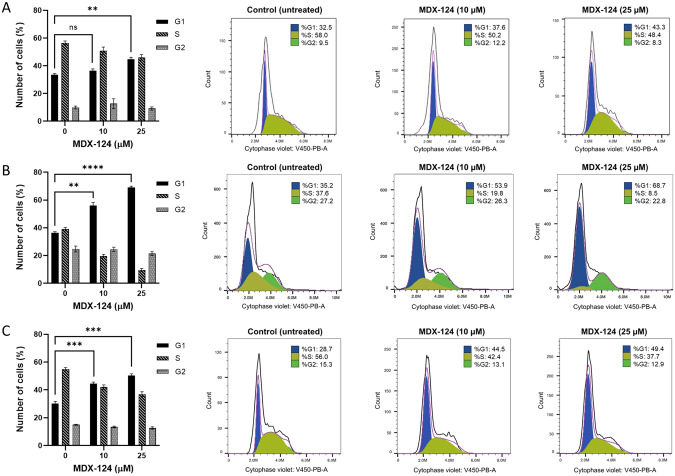
MDX-124 induces a G1 cell cycle arrest. **A** BxPC-3, **B** MDA-MB-231 and **C** A549 cancer cells (1 × 10^6^) treated with MDX-124 (10 and 25 µM) for 24 h. The percentage of cells in each phase of cell cycle (G1, S and G2) was assessed using CytoPhase™ Violet dye and quantified using the cell cycle module in FlowJo™ software and the Dean-Jett-Fox data fitting algorithm. The red line indicates the sum of all data modelled by FlowJo™ software, whilst the black line shows all of the raw data captured by the cell cycle gating parameters. Data are presented as the mean ± SEM (*n* = 3 experiments). Differences were compared using a 2-way ANOVA with Tukey’s multiple comparison correction where statistical significance indicated by ****p < 0.0001, ***p < 0.001 and **p < 0.01.

The cell cycle analysis was conducted using flow cytometry with pancreatic (BxPC-3), triple-negative breast (MDA-MB-231) and lung (A549) cancer cell lines. Following 24 h exposure to MDX-124 (10 and 25 µM) all cell lines had a significant dose-dependent increase in the percentage of cells in the G1 phase of the cell cycle when compared to untreated control cells (Fig. 2). Within the MDA-MB-231 cell line, the number of cells in G1 phase increased by 33.5% (from 35.2% to 68.7%) with a resulting decrease in S phase of 29.1% (from 37.6% to 8.5%) at 25 µM MDX-124 treatment. The A549 lung cancer cells showed a 21.2% increase in cells in G1 phase, with a resulting 18.3% decrease in S phase, and the BxPC-3 pancreatic cancer cells showed a 10.8% increase of G1 phase cells with a 10.4% decrease of cells in S phase, at 25 µM MDX-124 treatment. Consistent with the annexin V apoptosis assay, there was no evidence of a sub G0/G1 peak in cells treated with MDX-124 (Fig. 2). These data demonstrate that MDX-124 promoted a G1 phase cell cycle arrest but did not induce apoptosis.

### MDX-124 inhibits tumour growth in vivo

**Fig. 3:**
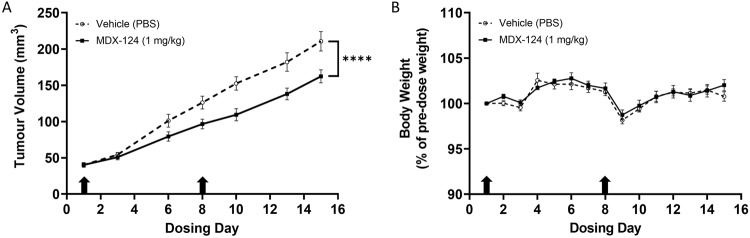
MDX-124 inhibits 4T1-luc triple-negative breast tumour growth in vivo. BALB/c mice (*n* = 12 per group) were inoculated orthotopically with 4T1-luc murine triple-negative breast cancer cells (5 × 10^4^) and randomised to receive either MDX-124 (1 mg/kg) or a vehicle control (PBS) treatment via intravenous injection on day 1 and day 8. **A** Tumour volume and **B** body weight measured at each corresponding time point. Arrows indicate day of dosing treatments received. Data are presented as the mean ± SEM with statistical significance calculated via mixed-effects model (REML) and indicated by ****p < 0.0001.

The 4T1-luc syngeneic mouse model of triple-negative breast cancer was utilised to assess the therapeutic efficacy of MDX-124 in vivo. Tumours were inoculated orthotopically and once reaching a suitable size, mice were randomised to receive either MDX-124 (1 mg/kg) or a vehicle control (PBS) on day 1 and day 8. MDX-124 treatment was found to significantly inhibit tumour growth versus the vehicle control treatment by day 8 and reached 23% growth inhibition by study end on day 15 (Fig. 3A). Additionally, MDX-124 treatment caused no noticeable change in animal body weight throughout the study (Fig. 3B), indicating it is well tolerated and has a favourable safety profile.

**Fig. 4:**
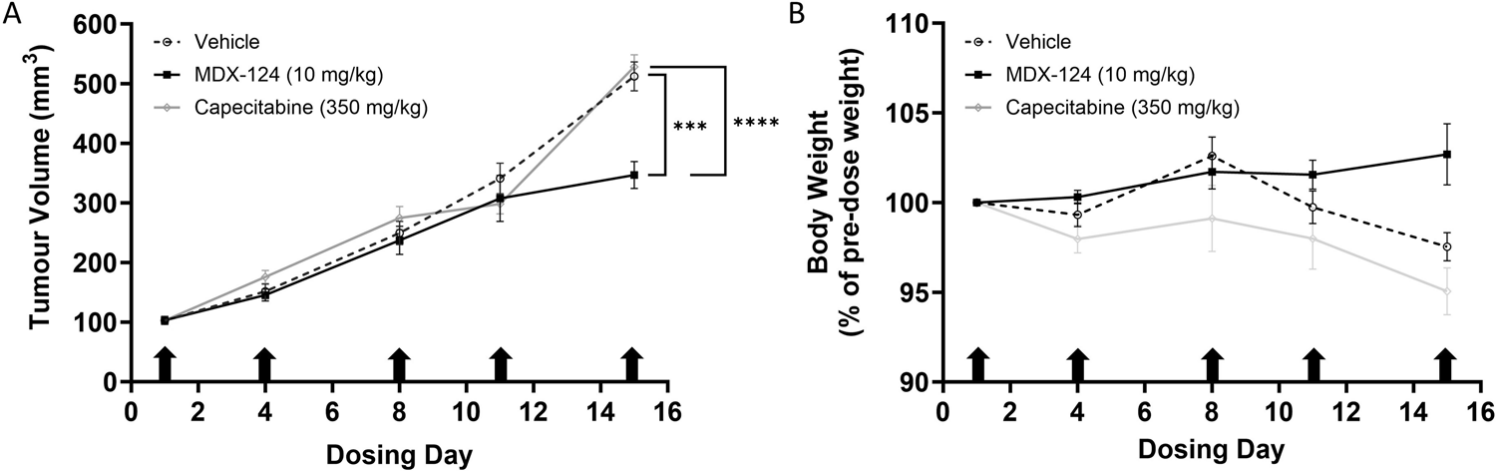
MDX-124 inhibits Pan02 pancreatic tumour growth in vivo. C57BL/6 mice (*n* = 30) were inoculated subcutaneously with 5 × 10^6^ Pan02 murine pancreatic cancer cells. Once tumours reached a volume of ~100 mm^3^ mice received an initial 13-day treatment phase of gemcitabine (80 mg/kg) and nab-paclitaxel (30 mg/kg) every 3 days for 4 doses. Mice were then randomised to receive either a vehicle control, capecitabine (350 mg/kg, daily) or MDX-124 (10 mg/kg, twice per week) as maintenance therapy for 14 days. **A** Tumour volume and **B** body weight were assessed three times per week. Arrows indicate day of dosing MDX-124 treatment received. Data are presented as the mean ± SEM with statistical significance calculated at dosing day 14 via an unpaired T-test indicated by ***p < 0.001 and ****p < 0.0001.

The efficacy of MDX-124 as a maintenance therapy was then evaluated in the Pan02 murine model of pancreatic cancer. Mice received a standard of care chemotherapeutic regimen of gemcitabine plus nab-paclitaxel for 13 days before entering the maintenance phase, at which point mice were randomised to receive either a vehicle control, capecitabine, or MDX-124. After 14 days of treatment, MDX-124 was found to significantly inhibit tumour growth when compared to both vehicle control and capecitabine (Fig. 4A). Additionally, MDX-124 treatment caused had no impact animal body weight throughout the study (Fig. 4B), indicating it is good tolerability in the maintenance setting.

## Discussion

ANXA1 activates FPR1 and FPR2 to initiate a complex network of intracellular signalling pathways that promote numerous cellular responses. Traditionally ANXA1 is considered a critical regulator of the inflammatory process, playing a role in both the innate and adaptive immune response, although its role in adaptive immunity is not as clear, possibly due to lower levels of ANXA1 expression on adaptive immune cells [30]. The anti-inflammatory effects of ANXA1 are mediated by glucocorticoids and lead to the resolution of inflammation through several mechanisms including limiting neutrophil recruitment, reducing secretion of pro-inflammatory molecules and enhancing the clearance of apoptotic cells by macrophages [5]. However, in addition to the well-documented role in immunity, there is significant evidence that ANXA1 is an important component of several upstream signalling pathways in a number of diseases [31]. In the context of cancer, ANXA1 has been shown to be involved in STAT3 [32], PI3K [33] and MAPK/ERK [11] signalling pathways which promote tumour initiation and progression.

In this present study we show that targeting ANXA1 with the humanised monoclonal antibody, MDX-124, can reduce cell growth in ANXA1-expressing cancer cells both in vitro and in vivo, providing further evidence that ANXA1 is a valid target for therapy in cancer. Using the MTT assay, we have shown that following a 72 h exposure to MDX-124 there is a consistent reduction in cellular metabolic activity resulting from a decline in cell viability across human cell lines derived from breast, ovarian, pancreatic, colorectal and some lung cancers.

This investigation corroborates several previous observations, including those which demonstrated ANXA1 expression in the metastatic TNBC cell line MDA-MB-231 was linked to enhanced cell proliferation and cell cycle transition [16] and that a decrease in cell proliferation was associated with G_1_ phase cell cycle arrest following ANXA1 knockdown via siRNA in pancreatic cancer cell lines [15, 34, 35].

To validate the efficacy of MDX-124 in vivo, a syngeneic model of TNBC was chosen. This tumour type is well-documented as expressing high levels of ANXA1 which correlates with poorer survival [15, 34]. Furthermore, since a syngeneic model has a fully functioning immune system, both direct and immune-mediated anti-tumour activity caused by ANXA1-targeted therapy can be observed. Tumour growth was found to be significantly inhibited after two weekly doses of MDX-124 at 1 mg/kg versus vehicle control-treated animals. Silencing of ANXA1 has been reported to reduce tumour growth in vivo in other studies where ANXA1 knockdown in SUM149 TNBC and HCT116 colorectal cancer cells grown as subcutaneous xenografts have been shown to grow significantly slower than non-silenced cancer cells [34, 36].

As there were no observed side effects or signs of toxicity related to dosing with MDX-124 in either the in vivo TNBC model described here or in our GLP NHP and rat toxicity studies (data not shown), a further study was performed to evaluate the efficacy of MDX-124 in the maintenance setting using the Pan02 syngeneic model of pancreatic cancer. Tumour growth was found to be significantly inhibited after two weeks of maintenance therapy in MDX-124 treated animals versus those receiving either a vehicle control or capecitabine (used clinically as maintenance therapy in several gastrointestinal cancers). Additionally, as with the TNBC model and GLP NHP and rat toxicity studies, no sign of toxicity attributable to MDX-124 were observed. This suggests that ANXA1-targeted therapy may have utility in tumour types like pancreatic cancer which rely on highly aggressive front-line chemotherapeutic treatment regimens.

Despite accumulating evidence surrounding the oncogenic roles of ANXA1, specifically targeting this protein in the context of therapy has its challenges. This is due, in part, to the high degree of homology amongst each member of the annexin protein family, particularly within the core domain [1]. Additionally, understanding ANXA1 biology is complex as it is susceptible to proteolytic cleavage in several physiological contexts including within tumour tissue and can be present in the nucleus, cytoplasm or extracellular environment [3, 7]. Indeed, assessing ANXA1 expression by immunohistochemistry has been shown to be complicated by a lack of available antibodies that are both specific for ANXA1 and can bind the cleaved form of ANXA1 previously identified in tumour vasculature [37]. The anti-ANXA1 antibody used in this study (MDX-124) binds specifically, with low nanomolar affinity, to ANXA1 via a unique discontinuous epitope within domain III of ANXA1 when in its activated Ca^2+^-bound conformation [29]. The binding specificity along with the anti-cancer activity demonstrated in this study suggests MDX-124 is a strong therapeutic antibody candidate for patients with tumours expressing high levels of ANXA1. This may be of particular benefit in treating clinically challenging cancers associated with poor 5-year overall survival rates, as shown by the Kaplan-Meier survival analyses presented here.

In summary we have conducted the first study to describe the mechanism of action of MDX-124, a humanised anti-ANXA1 monoclonal antibody. We demonstrated that in ANXA1-expressing breast, ovarian, pancreatic, colorectal and lung cancer cell lines, MDX-124 treatment suppressed cell proliferation. We also demonstrated that the reduced cell proliferation caused by MDX-124 was driven by a G1 phase cell cycle arrest and not through the induction of apoptosis. Furthermore, we substantiated our in vitro analysis and show that MDX-124 significantly inhibited tumour growth using in vivo syngeneic mouse models of both TNBC and pancreatic cancer.

Whilst this study successfully demonstrated MDX-124 has the potential to impact on ANXA1 overexpressing cancers, there were some limitations. There was a restricted quantity of antibody material available in some of the early experiments, in particular the annexin V apoptosis assay. Consequently, a lower maximal concentration of 5 µM MDX-124 was used rather the 10 µM MDX-124 used in other later experiments. Whilst we would have preferred to use the same maximal concentration across all the assay formats for consistency, it raises the possibility that we may have seen an impact on apoptosis if the higher concentration had been used. Similarly, dosing of MDX-124 in the in vivo TNBC mouse model was lower and less frequent than the dose regimen currently used, suggesting tumour growth inhibition in this model may be improved further.

Future studies will focus on exploring the effect of MDX-124 on other cancer-related processes shown previously to be driven by ANXA1 overexpression including invasion and migration, drug resistance and angiogenesis [17–19]. Interestingly, several earlier reports observed enhanced cell motility and reduction of cell polarity leading to epithelial-mesenchymal-transition (EMT), mediated in part by ANXA1-directed remodelling of the actin cytoskeleton [38]. In addition, interrogating the effect of MDX-124 on key oncogenic cell signalling pathways such as PI3K/AKT/mTOR or ERK could further delineate the mechanistic basis of cancer cell growth inhibition we have observed and could provide a rationale for selecting appropriate agents for use in combination therapy studies.

In conclusion this study provides evidence validating the idea that ANXA1-targeted therapy with MDX-124 could provide a novel and effective treatment option for patients whose tumours overexpress ANXA1. Furthermore, a First-In-Human study evaluating the safety and tolerability of MDX-124 alone and in combination with anti-cancer therapies has recently been initiated in patients with locally advanced, unresectable or metastatic solid malignancies known to overexpress ANXA1.

## Materials and methods

### Cell culture

A panel of human cancer cell lines were obtained from the European Collection of Authenticated Cell Cultures (ECACC) (Porton Down, UK) (Supplementary Table 1). Cell lines were incubated at 37 °C with 5% CO_2_ atmosphere and maintained as instructed by ECACC in culture media containing 10% foetal bovine serum (Life Technologies, Paisley, UK), 2 mM L-glutamine (Life Technologies, USA) and 100 U/ml penicillin and streptomycin (Sigma–Aldrich, Haverhill, UK). Any additional supplements are detailed in Supplementary Table 1.

### Antibodies

The humanised anti-ANXA1 antibody (MDX-124) used in this study was supplied by Medannex Ltd., (Edinburgh, UK). A monoclonal isotype control human IgG1 antibody was obtained from Thermo Fisher Scientific (Loughborough, UK).

### MTT assay

For adherent cell lines, cell suspensions were generated as previously described [39] and seeded into 96-well plates at densities varying between 5 × 10^3^ and 1 × 10^4^ cells per well, depending on the cell line. After 24 h, growth medium was removed and 100 µL of medium containing either MDX-124 or IgG1 isotype control at various concentrations (2.5–10 µM) was added in triplicate to the plate. For non-adherent cell lines, cells were re-suspended at a density of 1 × 10^5^ in medium containing either MDX-124 or IgG1 isotype control antibody (2.5–0 µM) and 100 µL was added in triplicate to the plate.

Plates were incubated at 37 °C for 72 h before 25 µL of 3-(4,5-dimethylthiazol-2-yl)-2,5-diphenyl tetrazolium bromide (MTT) (Thermo Fisher Scientific, UK) was added to all wells. Plates were then incubated at 37 °C for 2–4 h before 100 µL of 10% sodium dodecyl sulfate (SDS) in phosphate-buffered saline (PBS) was added to all wells. Plates were left in the dark overnight at 37 °C. Absorbances were measured for each well using a spectrophotometer (Tecan, Männedorf, Switzerland) at 492 nm, and background values measured at 620 nm were subtracted from the measurement.

Cell viability following exposure to either MDX-124 or the IgG1 isotype control antibody was calculated relative to untreated control cells. The responses of all cell lines to either treatment was determined by a minimum of three independent experiments.

### Multispectral imaging flow cytometry

Multispectral imaging flow cytometry was conducted using the ImageStream®^X^ Mark II system as described previously [40]. Images of >3000 cells were captured in brightfield illumination for ANXA1 protein localisation, Annexin V-FITC positive (apoptotic) cells, propidium iodide (late apoptotic/necrotic) and DRAQ5 staining of the nuclear region of each cell. Following excitation with a 488 nm laser at a power setting of 75 mW, all images were captured using a 60× objective lens using extended depth of field. Images of cells were acquired at a rate of approximately 50–100 cell images per second.

### Cellular localisation of ANXA1 by imaging flow cytometry

The localisation of ANXA1 within different cellular compartments (nucleus, cytoplasm, or membrane) was determined via imaging flow cytometry. Untreated cells or those exposed to MDX-124 (10 µM) for 72 h were washed in PBS, pH 7.4 (Severn Biotech, UK) and fixed in methanol and acetone (50:50 vol/vol) for 20 min at 4 °C. Following a further PBS wash, cells were permeabilised in a solution of PBS containing 0.5% (vol/vol) Triton™ X-100 (Sigma–Aldrich, UK). After blocking the cells in PBS containing 5% FBS, cells were incubated overnight at 4 °C in a 5 µM concentration of MDX-124 (primary antibody) in PBS containing 0.5% FBS using gentle agitation. Primary antibody was removed from the cells, followed by three washes in wash buffer (PBS containing 0.1% Triton^TM^ X-100) then incubation in goat anti-human (H + L) cross-adsorbed, Alexa Fluor™ 488 secondary antibody (Thermo Fisher Scientific, UK), diluted 1:1000 for 1 h at room temperature. Following three exchanges of wash buffer, cells were re-suspended in 50 µL PBS containing 1 µM DRAQ5™ DNA stain (Biostatus, UK) and subjected to image capture within 1 h. Image compensation was conducted as previously described [40]. Following the capture of >3000 cells, images were analysed using Ideas™ image analysis software. The localisation of ANXA1 in specific cellular compartments was quantified by creating a series of masks identifying these cellular regions. The average level of fluorescence attributed to ANXA1 protein was quantified.

### Cell cycle analysis

Aliquots of 1 × 10^6^ MIA PaCa-2, MDA-MB-231 and A549 cells were incubated for 24 h with 10 µM or 25 µM of MDX-124. Cells were harvested and labelled with 8 µM Cytophase™ violet dye. Cells were incubated at 37 °C for 30 min prior to analysis by flow cytometry (CytoFLEX LX, Beckman Coulter, USA). 10,000 events per sample were acquired and FCS files were subsequently analysed using the Dean-Jett-Fox cell cycle fitting algorithm in FlowJo™ v10.8 software (BD Life Sciences, USA) to determine the percentage of cells in each phase of the cell cycle.

### In vivo efficacy studies

In the 4T1-luc murine triple-negative breast cancer model, nine-week-old female BALB/c mice were inoculated orthotopically with 5 × 10^4^ 4T1-luc cancer cells. Once tumours reached a volume of ~50 mm^3^, mice (*n* = 12/group) were randomised to receive either MDX-124 (1 mg/kg) or a vehicle control (PBS) treatment via intravenous injection on dosing day 1 and day 8. Body weight and clinical signs were observed and recorded three times per week. Tumour volumes were assessed three times per week and calculated using the formula 0.5 (L × W^2^) by measuring the tumour in two dimensions using electronic callipers. In the Pan02 murine pancreatic cancer model, eight-week-old female C57BL/6 mice were inoculated subcutaneously with 5 × 10^6^ Pan02 cancer cells. Once tumours reached a volume of ~100 mm^3^, mice (*n* = 30) received an initial 13-day treatment phase of gemcitabine (80 mg/kg) and nab-paclitaxel (30 mg/kg) once every 3 days for 4 doses. Mice were then entered into a 14-day maintenance treatment phase and randomised to receive either a vehicle control (intravenous injection), capecitabine (350 mg/kg, daily via oral administration) or MDX-124 (10 mg/kg, twice per week via intravenous injection). Tumour volumes were assessed three times per week and calculated using the formula: Tumour volume (mm^3^) = (a × b^2^/2), where ‘b’ is the smallest diameter and ‘a’ is the largest diameter.

Animals were humanely sacrificed at the end of the dosing period or if they displayed any adverse clinical signs or loss of clinical condition.

### Statistical analysis

All statistical analyses were performed using Prism v9.4 software (GraphPad, USA). For MTT assays, data is presented as the mean ± SEM with a Mann–Whitney U-test used to determine any difference between treatment groups. For the 4T1-luc in vivo efficacy study, data is presented as the mean ± SEM with any statistically significant difference between treatment groups assessed using a mixed-effects model (REML). For the Pan02 in vivo efficacy study, data is presented as the mean ± SEM with any statistically significant difference between treatment groups assessed via unpaired T-test. For the survival analysis, a log-rank (Mantel-Cox) test was performed to determine any difference in survival between patients in lower and upper quartiles of ANXA1 expression. Statistical significance was set as follows: *p < 0.05, **p < 0.01, *** *p* < 0.001 and ****p < 0.0001.

### Supplementary information

Supplementary material

## References

[1] Gerke V, Moss SE (2002). Annexins: from structure to function. Physiol Rev.

[2] Perretti M, D’Acquisto F (2009). Annexin A1 and glucocorticoids as effectors of the resolution of inflammation. Nat Rev Immunol.

[3] Williams SL, Milne IR, Bagley CJ, Gamble JR, Vadas MA, Pitson SM (2010). A proinflammatory role for proteolytically cleaved annexin A1 in neutrophil transendothelial migration. J Immunol.

[4] D’Acquisto F, Piras G, Rattazzi L (2013). Pro-inflammatory and pathogenic properties of annexin-A1: the whole is greater than the sum of its parts. Biochem Pharm.

[5] Sugimoto MA, Vago JP, Teixeira MM, Sousa LP (2016). Annexin A1 and the resolution of inflammation: modulation of neutrophil recruitment, apoptosis, and clearance. J Immunol Res.

[6] Araújo TG, Mota STS, Ferreira HSV, Ribeiro MA, Goulart LR, Vecchi L (2021). Annexin a1 as a regulator of immune response in cancer. Cells.

[7] Boudhraa Z, Bouchon B, Viallard C, D’Incan M, Degoul F (2016). Annexin A1 localization and its relevance to cancer. Clin Sci.

[8] Bai XF, Ni XG, Zhao P, Liu SM, Wang HX, Guo B (2004). Overexpression of annexin 1 in pancreatic cancer and its clinical significance. World J Gastroenterol.

[9] Ydy LRA, do Espírito Santo GF, de Menezes I, Martins MS, Ignotti E, Damazo AS (2016). Study of the annexin A1 and its associations with carcinoembryonic antigen and mismatch repair proteins in colorectal cancer. J Gastrointest Cancer.

[10] Biaoxue R, Zhao C, Liu H, Ming Z, Cai X, Gao W (2014). Elevated serum annexin A1 as potential diagnostic marker for lung cancer: a retrospective case-control study. Am J Transl Res.

[11] Cheng TY, Wu MS, Lin JT, Lin MT, Shun CT, Huang HY (2012). Annexin A1 is associated with gastric cancer survival and promotes gastric cancer cell invasiveness through the formyl peptide receptor/extracellular signal-regulated kinase/integrin beta-1-binding protein 1 pathway. Cancer.

[12] Liu YF, Zhang PF, Li MY, Li QQ, Chen ZC (2011). Identification of annexin A1 as a proinvasive and prognostic factor for lung adenocarcinoma. Clin Exp Metastasis.

[13] Yom CK, Han W, Kim SW, Kim HS, Shin HC, Chang JN (2011). Clinical signifcance of annexin A1 expression in breast cancer. J Breast Cancer.

[14] Bhardwaj A, Ganesan N, Tachibana K, Rajapakshe K, Albarracin CT, Gunaratne PH (2015). Annexin A1 preferentially predicts poor prognosis of basal-like breast cancer patients by activating mTOR-S6 signaling. PLoS ONE.

[15] Gibbs LD, Vishwanatha JK (2018). Prognostic impact of AnxA1 and AnxA2 gene expression in triple-negative breast cancer. Oncotarget.

[16] Vecchi L, Alves Pereira Zóia M, Goss Santos T, de Oliveira Beserra A, Colaço Ramos CM, França Matias Colombo B (2018). Inhibition of the AnxA1/FPR1 autocrine axis reduces MDA-MB-231 breast cancer cell growth and aggressiveness in vitro and in vivo. Biochim Biophys Acta Mol Cell Res.

[17] Yi M, Schnitzer JE (2009). Impaired tumor growth, metastasis, angiogenesis and wound healing in annexin A1-null mice. Proc Natl Acad Sci USA.

[18] Belvedere R, Bizzarro V, Popolo A, Dal Piaz F, Vasaturo M, Picardi P (2014). Role of intracellular and extracellular annexin A1 in migration and invasion of human pancreatic carcinoma cells. BMC Cancer.

[19] Onozawa H, Saito M, Saito K, Kanke Y, Watanabe Y, Hayase S (2017). Annexin A1 is involved in resistance to 5-FU in colon cancer cells. Oncol Rep.

[20] Bai F, Zhang P, Fu Y, Chen H, Zhang M, Huang Q (2020). Targeting ANXA1 abrogates Treg-mediated immune suppression in triple-negative breast cancer. J Immunother Cancer.

[21] Moraes LA, Kar S, Foo SL, Gu T, Toh YQ, Ampomah PB (2017). Annexin-A1 enhances breast cancer growth and migration by promoting alternative macrophage polarization in the tumour microenvironment. Sci Rep.

[22] Weyd H, Abeler-Dörner L, Linke B, Mahr A, Jahndel V, Pfrang S (2013). Annexin A1 on the surface of early apoptotic cells suppresses CD8+ T cell immunity. PLoS ONE.

[23] Novizio N, Belvedere R, Pessolano E, Tosco A, Porta A, Perretti M (2020). Annexin A1 released in extracellular vesicles by pancreatic cancer cells activates components of the tumor microenvironment, through interaction with the formyl-peptide receptors. Cells.

[24] Geary LA, Nash KA, Adisetiyo H, Liang M, Liao CP, Jeong JH (2014). CAF-secreted annexin A1 induces prostate cancer cells to gain stem cell-like features. Mol Cancer Res.

[25] Wang LD, Yang YH, Liu Y, Song HT, Zhang LY, Li PL (2008). Decreased expression of annexin A1 during the progression of cervical neoplasia. J Int Med Res.

[26] Petrella A, Festa M, Ercolino SF, Zerilli M, Stassi G, Solito E (2006). Annexin-1 downregulation in thyroid cancer correlates to the degree of tumor differentiation. Cancer Biol Ther.

[27] Shen D, Nooraie F, Elshimali Y, Lonsberry V, He J, Bose S (2006). Decreased expression of annexin A1 is correlated with breast cancer development and progression as determined by a tissue microarray analysis. Hum Pathol.

[28] Fu Z, Zhang S, Wang B, Huang W, Zheng L, Cheng A (2020). Annexin A1: a double-edged sword as novel cancer biomarker. Clin Chim Acta.

[29] Gramlich M, Hays HCW, Crichton S, Kaiser PD, Heine A, Schneiderhan-Marra N (2021). HDX-MS for epitope characterization of a therapeutic antibody candidate on the calcium-binding protein annexin-A1. Antibodies.

[30] Gavins FNE, Hickey MJ (2012). Annexin A1 and the regulation of innate and adaptive immunity. Front Immunol.

[31] Kelly L, McGrath S, Rodgers L, McCall K, Tulunay Virlan A, Dempsey F (2022). Annexin‐A1: the culprit or the solution?. Immunology.

[32] Vecchi L, Mota STS, Zóia MAP, Martins IC, de Souza JB, Santos TG (2022). Interleukin-6 signaling in triple negative breast cancer cells elicits the annexin A1/formyl peptide receptor 1 axis and affects the tumor microenvironment. Cells.

[33] Khau T, Langenbach SY, Schuliga M, Harris T, Johnstone CN, Anderson RL (2011). Annexin-1 signals mitogen-stimulated breast tumor cell proliferation by activation of the formyl peptide receptors (FPRs) 1 and 2. FASEB J.

[34] Johnstone CN, Tu Y, Langenbach S, Baloyan D, Pattison AD, Lock P (2021). Annexin a1 is required for efficient tumor initiation and cancer stem cell maintenance in a model of human breast cancer. Cancers.

[35] Liu QH, Shi ML, Bai J, Zheng JN (2015). Identification of ANXA1 as a lymphatic metastasis and poor prognostic factor in pancreatic ductal adenocarcinoma. Asian Pac J Cancer Prev.

[36] Rubinstein MR, Baik JE, Lagana SM, Han RP, Raab WJ, Sahoo D (2019). Fusobacterium nucleatum promotes colorectal cancer by inducing Wnt/β‐catenin modulator Annexin A1. EMBO Rep.

[37] Allen KL, Cann J, Zhao W, Peterson N, Lazzaro M, Zhong H (2020). Upregulation of annexin A1 protein expression in the intratumoral vasculature of human non–small-cell lung carcinoma and rodent tumor models. PLoS ONE.

[38] De Graauw M, Van Miltenburg MH, Schmidt MK, Pont C, Lalai R, Kartopawiro J (2010). Annexin A1 regulates TGF-β signaling and promotes metastasis formation of basal-like breast cancer cells. Proc Natl Acad Sci USA.

[39] Plumb JA. Cell Sensitivity Assays: The MTT assay. In: Cancer cell culture. New Jersey: Humana Press; 2004. p. 165–70.

[40] Parris CN, Adam Zahir S, Al-Ali H, Bourton EC, Plowman C, Plowman PN (2015). Enhanced γ-H2AX DNA damage foci detection using multimagnification and extended depth of field in imaging flow cytometry. Cytom Part A.

