# Supplementary material for: A therapeutic antibody targeting annexin-A1 inhibits cancer cell growth in vitro and in vivo
Source: Oncogene. 2024 Jan 10;43(8):608–14. doi: 10.1038/s41388-023-02919-9 (PMC10873194; doi:10.1038/s41388-023-02919-9)
Supplement: Supplementary file 1 — Supplementary material [file 41388_2023_2919_MOESM1_ESM.docx]

**SUPPLEMENTARY INFORMATION**

**A novel therapeutic antibody targeting annexin-A1 inhibits cancer cell growth *in vitro* and *in vivo*.**

Hussein N Al-Ali^1^, Scott J Crichton^2^, Charlene Fabian^2^, Chris Pepper^3^, David R Butcher^1^, Fiona C Dempsey^2^ and Christopher N. Parris^1^.

1. Anglia Ruskin University, School of Life Science, Faculty of Science and Engineering, East Road, Cambridge, UK, CB1 1PT
2. Medannex Ltd, 1 Lochrin Square, 92-98 Fountainbridge, Edinburgh, Scotland, UK, EH3 9OA
3. Brighton and Sussex Medical School, Medical Research Building, Falmer, Brighton, UK, BN1 9PX

**Contents:**

Supplementary Materials and Methods

Supplementary Table

Supplementary Figures (1-3)

**Supplementary Materials and Methods**

**ANXA1 Expression and Survival Analysis**

ANXA1 gene expression and probability of survival data from the Cancer Genome Atlas (TCGA) for breast, pancreatic, ovarian, lung and colorectal cancers were accessed and downloaded via the University of California Santa Cruz Xena platform (Goldman *et al*., 2020). Kaplan-Meier survival plots datasets were generated from the each TCGA dataset using Prism v9.4 software (GraphPad, USA).

**Annexin V Apoptosis Assay**

Measurement of apoptosis in MDX-124 treated and untreated cancer cells was performed using an Annexin V-FITC apoptosis staining/detection kit (Abcam, Cambridge, UK). In brief, 1 x 10^5^ MCF-7 or Caco-2 cells were added to individual wells of a 12-well microtiter plate. Following 24 h incubation to allow for cell attachment, cells were exposed to 5 µM MDX-124 for 72 h. Thereafter, cells were harvested and resuspended in 50 µL binding buffer prior to the addition of 5 µL Annexin V-FITC and 5 µL propidium iodide to the cells. Untreated and MDX-124 exposed cells were incubated at room temperature for 5 min and images of at least 3000 cells were captured by imaging flow cytometry.

**Supplementary Tables**

**Supplementary Table 1**. List of human cancer cell lines and culture media

| **Cell Line** | **Description** | **Culture Media** |
| --- | --- | --- |
| **MCF-7** | Breast (luminal A) | DMEM |
| **MCF-7/TAMR7** | Tamoxifen resistant MCF-7 subline | Phenol red-free DMEM: Ham’s F12 + 1 µM tamoxifen |
| **HCC1806** | Breast (TNBC) | DMEM |
| **MDA-MB-231** | Breast (TNBC) | DMEM |
| **A2780** | Ovarian | RPMI |
| **A2780ADR** | Adriamycin resistant A2780 subline | RPMI + 100 nM adriamycin |
| **A2780cis** | Cisplatin resistant A2780 subline | RPMI + 1 µM cisplatin |
| **NCI-H69** | Small cell lung carcinoma | RPMI |
| **NCI-H69/CPR** | Cisplatin resistant NCI-H69 subline | RPMI + 1 µM cisplatin |
| **COR-L23** | Large cell lung carcinoma | RPMI |
| **COR-L23.5010** | Multi-drug resistant COR-L23 subline | RPMI + 1 µg/mL adriamycin |
| **A549** | Lung non-small cell lung cancer | Ham’s F12 |
| **HCT116** | Colon carcinoma | McCoy’s 5A |
| **SW480** | Colon adenocarcinoma | L-15 |
| **Caco-2** | Caucasian colon adenocarcinoma | MEM + 1% NEAA |
| **MIA PaCa-2** | Caucasian pancreatic carcinoma | DMEM |
| **BxPC-3** | Primary pancreatic adenocarcinoma | RPMI |
| **PANC-1** | Caucasian pancreas (undetected Y chromosome) | DMEM |

**Supplementary Figures**

**
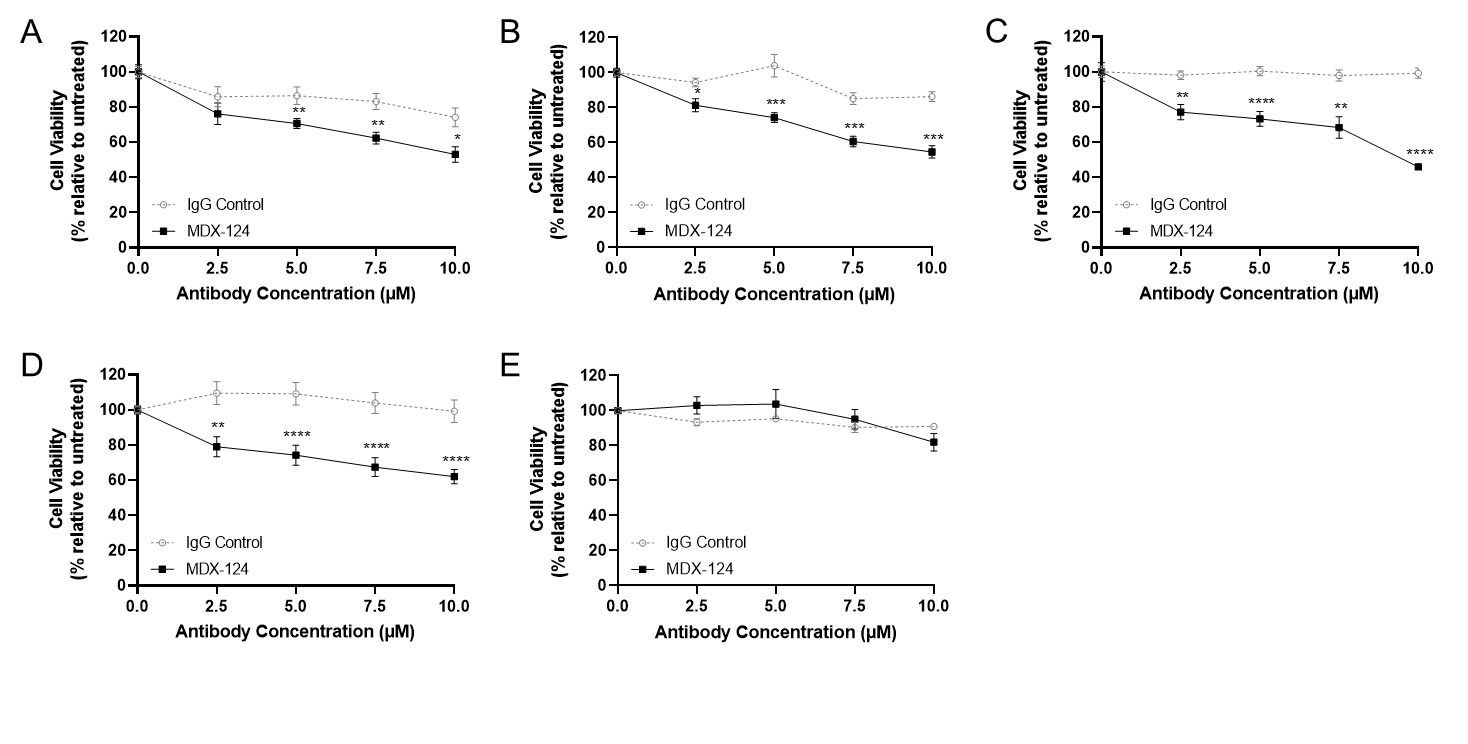
**

**Supplementary Figure 1.** **MDX-124 significantly reduces cancer cell proliferation.** (A) MCF-7/TAMR7, (B) PANC-1, (C) HCT116, (D) SW480 and (E) COR-L23.5010 cancer cell lines were treated for 72 h with either MDX-124 or an IgG isotype control (2.5, 5, 7.5 or 10 μM). Cell viability measured via MTT assay. Data are presented as the mean ± SEM of at least 3 independent experiments. Statistical significance calculated via Mann Whitney U-test and indicated by ****p<0.0001, ***p<0.001, **p<0.01 and *p<0.05.

**
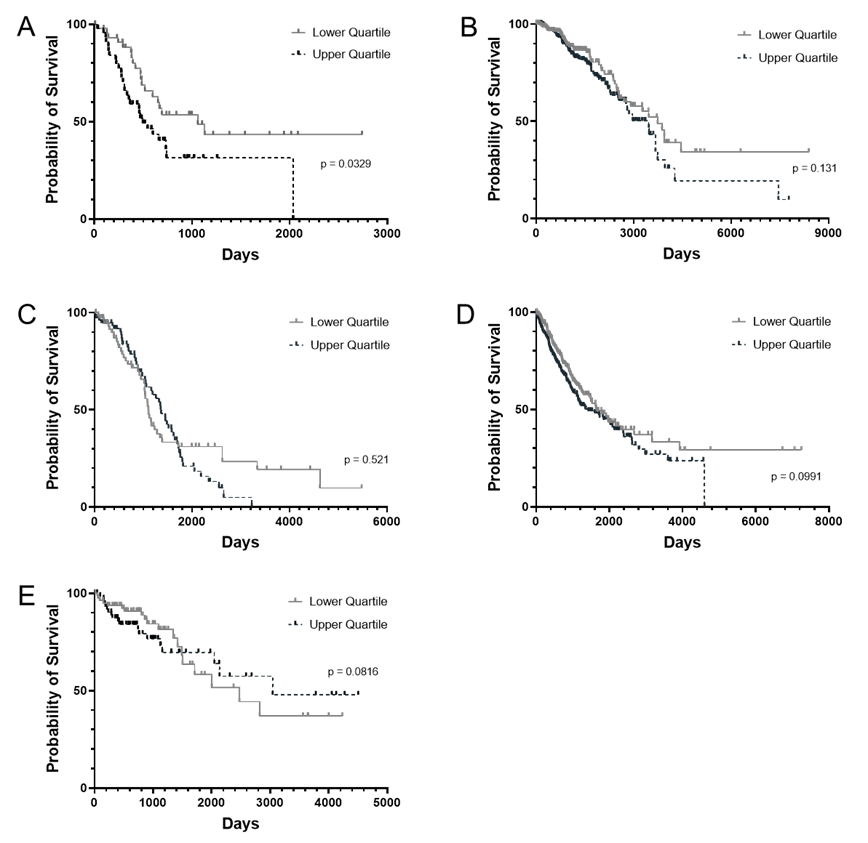
**

**Supplementary Figure 2. Higher ANXA1 expression correlates with poorer probability of survival in several cancer indications**. Kaplan-Meier survival plots for (A) pancreatic (n = 91), (B) breast (n = 596), (C) ovarian (n= 153) (D) lung (n = 538) and (E) colorectal (n = 160) cancer patients based on upper and lower quartile ANXA1 gene expression data obtained from The Cancer Genome Atlas. Differences in survival were compared using a logrank (Mantel-Cox) test where statistical significance was set at *p < 0.05.

**
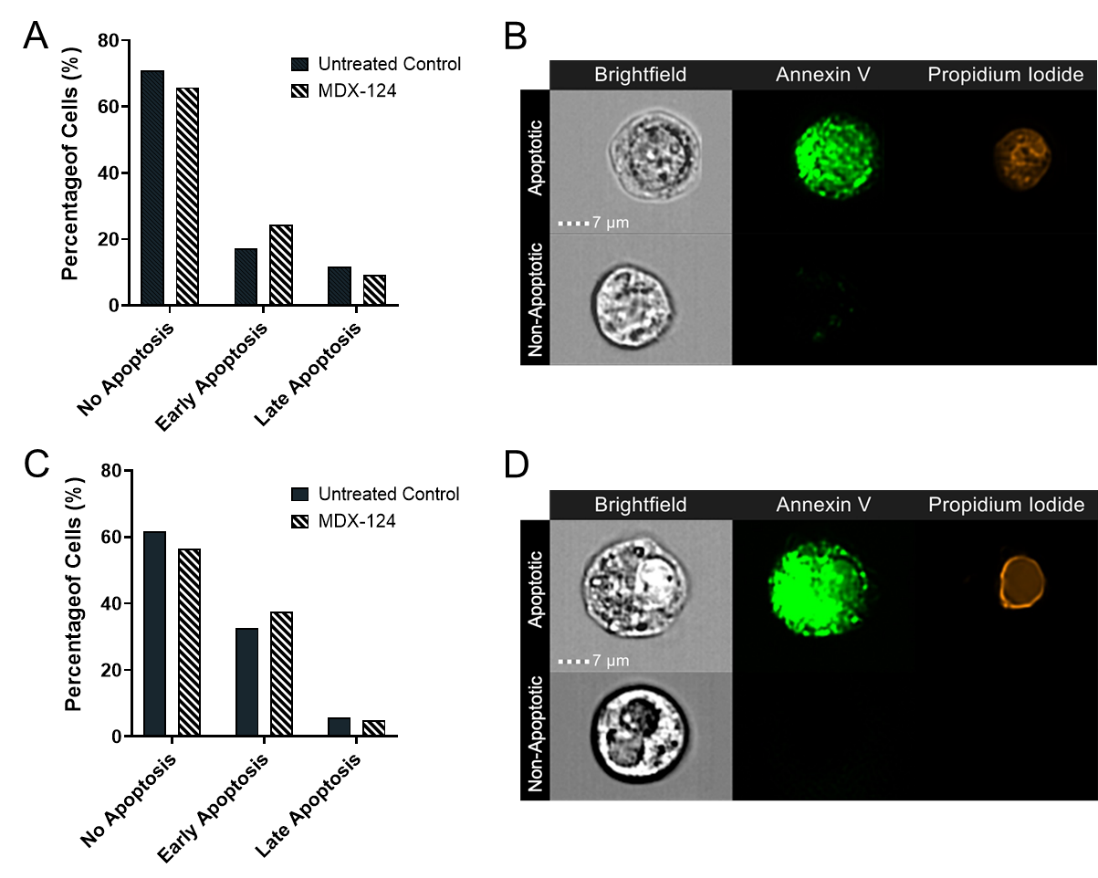
**

**Supplementary Figure 3. MDX-124 does not induce apoptosis in cancer cell lines**. Percentage of non-apoptotic, early apoptotic (annexin V membrane staining) and late apoptotic (annexin V and propidium iodide internalised) (A) MCF-7 and (C) Caco-2 cancer cells either untreated or treated with MDX-124 (5 µM) for 72 h. Representative examples of apoptotic and non-apoptotic (B) MCF-7 and (D) Caco-2 cancer cells taken by IFC.
